# Product News

**DOI:** 10.1155/S1463924680000351

**Published:** 1980


### Laboratory Organisation and Management

*F. Grover and P. Wallace*
*Butterworths Ltd., 1979, pp 241, £5.95, ISBN 408707933*

The authors of this useful book are experienced in laboratory management and safety aspects of medical laboratories and, although their specialism is to some extent reflected in the selection of material, most of the content is of general applicability. There are seven main chapters, Laboratory Planning and Layout, Selection and Management of Staff, Purchasing and Financial Control, Management of Stores, Laboratory Administration, Service Departments and Special Purpose Rooms, and Health and Safety. In addition there are brief chapters on Maintenance of Laboratory Premises and Equipment, Automation in the Laboratory and Management Techniques and Functions. Automation, including the use of computers, is covered in a mere six pages, and although most of the managerial and organizational problems are alluded to, the treatment is inevitably superficial and of rather limited value. There remains a real need for an in-depth evaluation of this complex and rapidly expanding subject area.

The book is intended to provide practical guidance and this indeed is its virtue. The responsibilities of a laboratory manager and the flexibility of approach accorded to him vary widely with the nature and size of the organization he serves. For example, laboratory managers in a large organization are unlikely, however much they might wish to do so, to be able to react to the content of the chapters on staff selection and purchasing and financial control; procedures for such matters will be enshrined in the protocols of the establishment and their amenability to modification, at least in the short term, will be minimal. On the other hand the chapter on health and safety aspects should prove useful to all laboratory managers.

Although primarily intended for laboratory managers, the book has a potentially wider readership. No young scientist intent on a laboratory career should embark on it in ignorance of the essential organizational procedures which exist to maintain a safe and efficient working environment, and a few hours with this book will be time well spent.

At current prices the book is good value, the style is straight forward and pleasantly free from the loftiness which so often attends those works exhorting sound and sensible behaviour.

*J.K. Foreman*

### Product News

### Titration systems

Radiometer have announced the introduction of the DTS885 and DTS886 white liquor titration systems for the automatic analysis of white, green and black liquor. The DTS885 system is for single sample determinations and the other system for on-line determinations. The titration process is microcomputer-controlled with the automatic addition of formaldehyde during the titration. All three equivalence points are automatically printed-out together with the calculated amounts of effective, active and total alkali as well as percent causticity and percent sulphidity. The range of analysis is 30-190 g NaOH/1 total alkali, with a repeatability of 0.4g NaOH/1 total alkali.

*Radiometer A/S, Emdrupvej 72, DK-2400 Copenhagen NV, Denmark.*

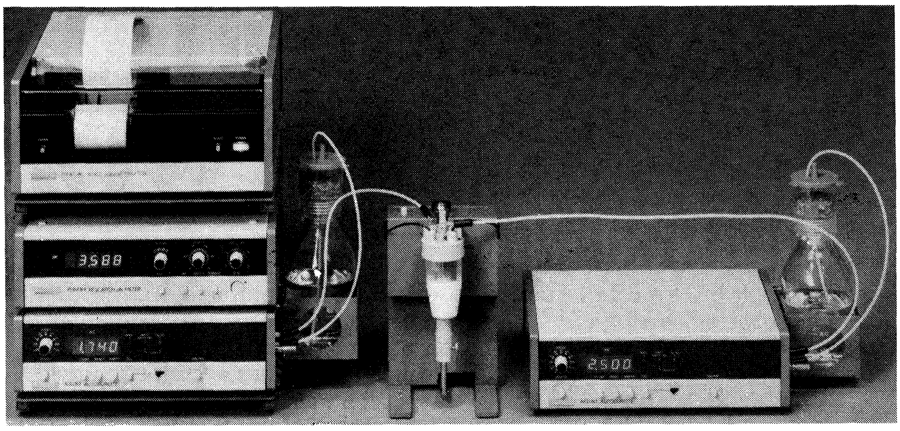

*White liquor automatic titration system from Radiometer*

### Fluxer for sample preparation

The Claisse Fluxer VI is an instrument to make fusions in the most effective way possible. It also makes casting and, depending on the container for casting, the end product is a glass disk if the glass is poured into a mould; or a solution if the glass is poured into a beaker containing an acid solution, following the method of Crow and Connolly. The design has been modified slightly so that one kind of Fluxers can make glass disks and another kind of Fluxers can make glass disks and solutions. In the latter, changing from one function to the other is done by moving one part only from one slot to another slot. It is even possible to make solutions and glass disks in the same time. For easy access to holders and samples at the rear of the instrument, the column can be rotated; it locks at one position for processing.

The fluxer can make any size of glass disks up to about 40 mm diameter; the size of the disks depends on the size of the moulds used.

*Claisse Scientific Corp, 7 Peace Jardins Merici Suite 1301, Quebec, G154N8 Canada.*

### Total carbon analyser

Techmation can now offer the Model 1258 Total carbon/total organic carbon analyser. A single automatic analysis cycle, of five minutes duration, involves inlet of a separate sample for each channel. Total carbon analysis is performed by aspiration of the first sample directly into an all-ceramic valve, which injects the sample into a high temperature reaction chamber. Quartz reaction tubes are suitable for most sample types. All carbon species are converted to carbon dioxide which is transferred by the carrier gas through a water-filled scrubber. This removes corrosive impurities and interferences to a non-dispersive infra-red analyser. The amplified signal from the NDIR is displayed on a recorder as a peak whose height corresponds to a specific carbon value.

For total organic carbon analysis, a second sample is automatically mixed with acid at a constant sample-to-acid ratio. Inorganic carbon is converted to carbon dioxide, which is removed from the system by automatic purging with nitrogen (99.5% efficiency). The sample which then contains only organic carbon is then analysed as before. Results are displayed on an indicating meter and recorder.

*Techmation Ltd, 58 Edgware Way, Edgware, Middx HA8 8JP, UK.*

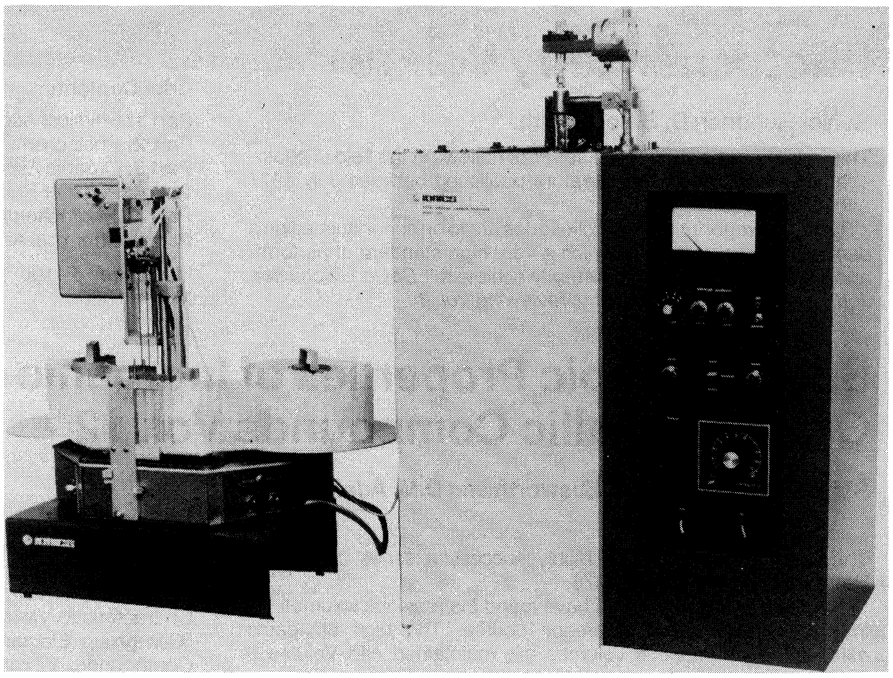

*Techmation's total carbon analyser*

### Mini-disk storage

The Tri-data Flexifile 21 from Wessex Electronics is a microprocessor-controlled mini-disk system offering the user flexibility in application and configuration. The product is designed for reliable information storage and communications. It is supported by proven disk electronics and software for data entry, editing and communications software.

The Flexifile 21 connects to a CRT or terminal printer transforming it into a workstation with intelligence, local storage and on-line communications capabilities. Data entry and edit operations are conducted off-line and stored on the mini-diskette. When data entry is completed, information stored on the diskette can be sent at high speeds to a remote processor. Data being received may be stored on the disk as well as being printed on the terminal.

*Wessex Electronics Ltd, 114-116 North Street, Downend, Bristol BS16 5SE, UK.*

### HPLC system

The Micromeritics 7500 system for high performance liquid chromatography is available from Coulter Electronics in both gradient and isocratic models. The microprocessor based 740 control module is the key to the system. To initiate analysis all operating parameters are entered via a keyboard. An alphanumeric display continually informs the operator of entry status. An analysis report is produced on a printer/plotter located in the control module. In addition to a chromatogram, the report contains details of gradient solvent conditions, flow rate pressure, temperature and operational status. An optical data reduction accessory is available which prints complete report information giving sample retention times, peak area and height percentage of concentration, sample and injection numbers.

Other system components include a new ternary solvent mixer Model 753 for more reliable low pressure solvent blending, a precision heated column compartment, a universal injector, variable and fixed wavelength detectors and a refractive index monitor.

*Coulter Electronics Ltd, Coldharbour Lane, Harpenden, Herts, UK.*

### Multipen plotter

An intelligent multipen digital plotter which can provide high quality technical drawings has been launched by Pye Unicam. The Phillips PM 8151 offers a programmable choice of eight pens.

Microprocessor control allows linear interpolation of vector generation, absolute and relative plotting as well as circles and arcs. Up to 120 different characters can be printed using five different fonts. Plotting area is 280 x 338 mm with accuracy of 0.1% full scale and linearity better than ± 0.1%. Grid marking and x- and y-axis scaling are possible.

Either V24 serial or IEC 625/IEEE 488 bus interfacing is available. An 800 byte buffer, optionally extendable by another 1 Kbyte buffer, provides storage for incoming data. A ROM expansion allows addition of special characters as well as user-defined subroutines to the standard available software.

Operation is controlled by a Zilog Z 80 microprocessor for simple operation and programming. The microprocessor also monitors all actions to ensure maximum drawing quality. Self test at the power on stage ensures correct functioning of the instruments.

The unit operates on 110, 220 or 240 V ac ± 10% at 50 or 60 Hz with power consumption about 30 VA.

*Pye Unicam Ltd, York Street, Cambridge CB1 2PX, UK.*

### Hydrocarbon gas analyser

Techmation have announced the introduction of a new real-time hydrocarbon gas analyser from Maloy. The HC 500-2C is built around the design of the HC 500, but in addition to total hydrocarbon readings also measured methane and non-methane components. The analyser uses a flame ionisation detector, designed for stable operation in the absence of a source of clean combustion air. It does not require an air support module or filter/dessicant system. An internal sample pump is supplied.

Sample air is introduced directly into the FID to yield the total hydrocarbon reading. The pneumatic system then passes sample air through a catalytic oxidiser to the detector. The catalytic oxidiser converts all non-methane hydrocarbons to a non-detectable species, so only methane is detected. A reading of the non-methane component is obtained by electronic substraction. The minimum detection limit is 0.1 ppm methane.

*Techmation Ltd., 58 Edgware Way, Edgware, Middx HA8 8JP, UK.*

### Programmable analysis system.

The Digichem 4000 series is a fully automated wet chemical analysis system which can be either user pre-programmed or provided pre-programmed to perform a wide range of chemical tests for inorganic, reactive organic or metallic spaces at levels from ppb to concentrated solutions. It can perform a large range of EPA, ASTM or other standard methods. A precision titrator, a colorimeter and a selective ion analyser form the basis of the system. Single and multiple end point titrations can be performed with potentiometric redox or colorimetric end point detectors. An interference corrected single-beam dual wave length detector is available for colorimetric analysis. Selective analysis can be automatically performed using plot titrations or using standard addition techniques.

The system, which is designed in a modular expandable format, can analyse up to 200 samples using up to five analytical reagents using one or more of the analytical techniques described above. Automatic calibration and systems verification subroutines and automatic sample handling capabilities allow the instrument to be easily used and readily maintained. The computer compatible output interfaces are provided to link the Digichem 4000 into central data acquisition systems.

*Ionics Incorporated, 65 Grove Street, Walertown Massachussetts, 02172, USA.*

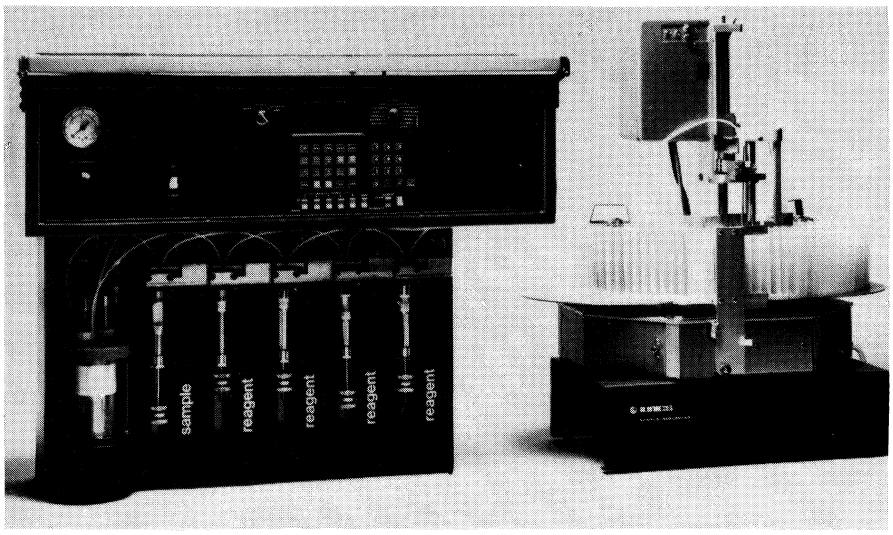

*Digichem 4000 programmable analysis system*

### Automatic concentrator

The CDS 320 Trapping Concentrator is a second-generation microprocessor-controlled instrument for concentrating trace organics from air, water, or solids and injecting them automatically into any GC, GC/MS, or GC/IR.

Incorporating the purge-and-trap technique, the CDS 320 is a compact system that employs advanced microprocessor technology to simplify its operation. For analytical procedures that permit it, the 320 automates the entire process: concentration and injection into a GC. The operator need only enter the desired parameters on the 320's easy-to-use keyboard and push the Start button. From that point on, the microprocessor takes over the responsibility for the analysis, insuring that it will be carried out precisely and consistently.

The CDS has many advanced features which increase its flexibility in operation. This flexibility is aptly illustrated by reference to the operational sequence for dual-trap CDS 320 interfaced to an automated GC shown below. Once the sample has been loaded into the appropriate container, the concentrator is started. This cycle can be repeated. The volatile organics are sparged (purged) for a specified time onto a trap, where they are absorbed and concentrated. Once the purge/desorb step is completed, the 320 waits for the GC ready signal. When the GC is ready, the instrument rapidly heats and backflushes the concentrate on the trap onto the GC column as a sharp pulse. The start of the GC program then is triggered by the instrument, by remote control. At the end of the GC analysis the trap can be reconditioned by entering into the bake mode, backflushing the trap, and venting the effluent at an elevated temperature. For continuous operation (traps A and B), the second trap is available for another sample collection while the first sample desorbs into the GC for analysis. Provision also has been included for independent operation of each trap (A only or B only).

*Chemical Data Systems, Inc. Oxford, Pennsylvania 19362, USA.*

### Scanning spectrophotometer

Easy operation, fast training, versatile outputs and sampling, superior data quality, and low cost are some of the features claimed for the Spectronic 2000 UV-visible double-beam spectrophotometer introduced by Bausch & Lomb.

A built-in microprocessor permits centralizing operating controls of the 2000 in a simple, functionally designed keyboard. Accessories, which include x-y and strip chart recorders, are also keyboard-controlled. The operator merely keys in any modifications to preprogrammed test parameters, and reads results on LED displays, English-language printouts and chart scans. Test parameters are printed out automatically. Details of setup and operation are handled automatically by the microprocessor, minimizing operator errors, reducing test time, and relieving the tedium of routine tasks.

The 2000 provides scans in %T, A, C, and first and second derivative of A. Also possible are time-rate and routine fixed-wavelength analyses. A complete array of data-enhancement features aids the analyst in quickly extracting the exact kind of required information from any scan – even from turbid samples. Included are automatic peak detection; variable signal averaging, scan rate, chart speed, delta lambda, scale range, and offset. More important is automated baseline storage, which nulls out anomalies arising from source-lamp-energy/wavelength variations, photodetector non-linearity and cuvette mismatch.

*Bausch & Lomb, Analytical Systems Division, 820 Linden Avenue, Rochester, N.Y. 14625, USA.*

### Dual-channel waveform digitiser

A dual-channel waveform digitiser with advanced signal-acquisition facilities and full systems capability is available from Tektronix. The instrument, the 7612D, has a 200 MHz sampling rate (8-bit resolution) a formattable 2048-word memory and a switchable sampling rate which can be changed up to 13 times within each record, is fully programmable, and is compatible with most general purpose interfaces.

It is designed to acquire two channels of analogue information at a bandwidth of up to 90 MHz, digitise the information and store it in the internal memory. The digitiser has two time bases, each of which can have different settings.

The analogue/digital convertor used in the digitiser uses an electron-beam/photodiode-array technique to produce a continuous 8-bit Gray-code representation of the analogue input.

Tektronix supplies this digitiser as part of its WP3000 series of signal processing systems, where the digitiser is used in conjunction with an appropriate computer, peripherals and software. Applications of the 7612D and allied systems are in complex waveform analysis or in the extensive or repetitive processing of waveforms.

*Tektronix UK Ltd, Beaverton House, PO Box 69, Harpendon, Herts, UK.*

### Solution handling system

FiAtron Systems have introduced an advanced, microprocessor controlled Solution Handling System SHS-200. Many repetitive analytical solution handling tasks can be readily automated with the SHS-200. The unit is designed to significantly upgrade the analytical productivity of any laboratory for either low or high volume workloads. The FiAtron SHS-200 can be readily interfaced with most analytical instruments.

In the flow injection modes, the SHS-200 injects either a fixed (100 $\mu$l) or a programmable volume of sample or reagent into a carrier stream. The carrier stream transports the sample toward the detector. Rates and volumes of injection as well as the number of repetitive injections per sample are under software control and are programmable from the front panel.

The system accommodates a wide range of analytical solution handling for a wide range of techniques in addition to its flow injection modes. The unit, which is a compact 17" wide x 8" high x 21" deep, can be readily linked from their sampler unit, the SHS-100, which has a 210 sample capacity. Other samplers can also be interfaced with the SHS-200.

*FiAtron Systems Inc, 7315 South First Street, 2 Oak Creek, 53154, USA.*

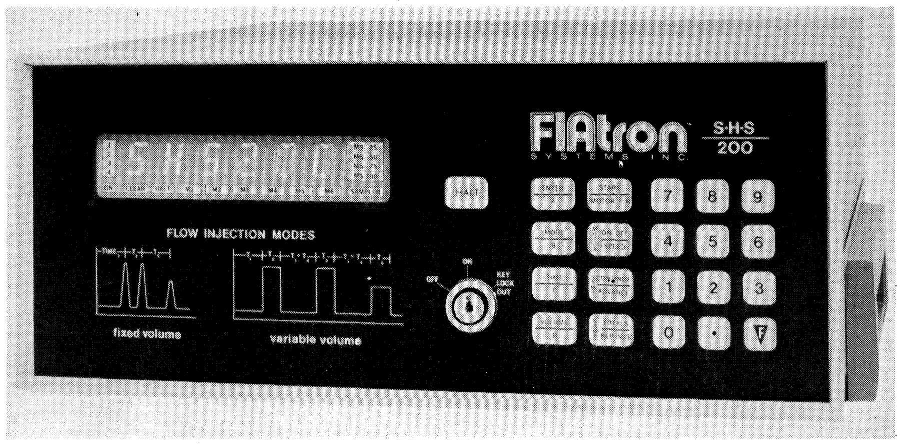

*FiAtron solution handling system*

### Autosampler

The PHOTOchem Autosampler was introduced at the Pittsburgh Conference. It has a precision sample transfer pump which, via pushbutton control, can be programmed to enter sample sizes of 1 ml, 10 ml,or 100 ml, into the analyser. A second pump dispenses the exact volume of reagents required for each analysis. Error due to sample carry-over is eliminated by the automatic cleaning and rinsing of all sample-carrying surfaces between determinations.

The sample carousel is a reliable cam and shuttle mechanism which accepts 11 removable, three-position tube racks. A special colour-coded tube rack activeates the unit's automatic stop feature after the required number of tests have been completed, up to the maximum of 33 determinations. The vertical-action sample sipper is designed to pierce Parafilm, or similar laboratory film, so that samples may be protected from atmospheric contamination, if desired.

Other features of the autosampler include a digital LED display which shows the number of the sample being analysed, a built-in drum printer which provides a permanent record of each analysis with sample number and concentration in parts per million, and choice of automatic or manual modes of operation.

*Barnstead Co, Division of Sybran Corp, 225 Rivermoor St, Boston, Ma 02132, USA.*

### Capillary viscometry systems

Brinkman Instruments have introduced a fully automatic capillary viscometry system. The Lauda Viscotimer S permits the simultaneous analysis and printout of up to six different samples, at different temperatures (if necessary). The smaller Lauda Viscoboy operates with one viscometer and uses a digital display rather than a printer.

Unique features of the system include a printout of sample identification and time; automatic compensation to permit use with transparent, non-transparent and tailing test liquids. Each channel has separate controls for setting of delay times, pumping pressure and number of tests per sample. An optional programmable calculator provides printout of sample no., time(s), mean, standard deviation and absolute or relative viscosity; standard accessories including see-through baths for 2,4 or 6 viscometers and a fully automatic washing apparatus for up to ten viscometers.

*Brinkman Instruments Subsidiary of Syvaren Corp, Cantiague Road, Westbury, New York, 11590, USA.*

### Computer, flame atomization system

A high performance flame atomization system coupled to a powerful computer has been announced by Varian. The AA-875 atomic absorption spectrophotometer promises to be a real boon to the analyst.

The new flame atomization system combines a precision engineered, adjustable nebulizer with a redesigned spray chamber, resulting in a demonstrably more efficient method of delivering the sample to the flame. The nebulizer, spray chamber and burners are mounted in a new adjustment assembly, providing smooth, tri-axial positioning.

The AA-875 microcomputer system provides the analyst with great flexibility in capturing and manipulating data. Photometric measurements are made in real time, providing improved accuracy, drift compensation, and background correction, as well as enhanced accuracy when tracking rapid transient signals encountered in flameless measurements. Up to five standards can be used to define a calibration curve. Calibration values can be obtained via a statistic function which takes the mean value of a series of discrete measurements.

The microcomputer also controls and coordinates the transfer of data from the instrument to the outside world, in both IEEE-488 and RS-232C formats. The external computer can provide flexible report generation, storage, recall and setup of key instrument parameters, and processing a display of primary absorption signals.

A number of options, such as the multiple lamp, background correction, automatic gas control, and automatic sample turntable can be added to the AA-875.

*Varian Instrument Group, 611 Hansen Way, Palo Alto, CA 94303, USA.*

### Sample application device

A sample application device from Camag has been designed for the application of small sample quantities (less than 5 $\mu$l) onto HPTLC layers. It is also useful when applying large sample volumes onto classical layers. The device, the Nanomat, will accommodate disposable glass capillaries for classical TLC layers, the Pt-Ir HPTLC application capillary or fixed volume nanopipettes for HPTLC layers. The capillary pipette is fitted with a steel sleeve and filled with sample solution. The pipette is inserted into the magnet head wherein it is suspended with its orifice a few millimetres above the layer. Upon actuating a release button the capillary lowers onto the layer surface. After a preselected time interval, the capillary automatically returns into its suspended position from where it is removed and refilled. The lowering speed can be regulated to avoid damage to the layer.

*Baird & Tatlock (London) Ltd, PO Box 1, RM1 1HA, UK.*

### Automated batch analyser for water chemistry

The Coulter Industrial Kem-O-Lab is an automated, water chemistry analyser designed for the busy laboratory. Up to six tests may be run at the same time on each set of samples. The most important feature is that it can operate continuously with very limited attention.

The system will analyse up to 240 tests per hour, and currently 12 analytical methods have been developed for water analysis. The unit operates unattended and provides output either as a digital printout or can be linked to an external computer.

The Kem-O-Lab is economical to operate. It performs only those tests requested and uses only the reagents necessary for the tests performed. Reagent consumption per test is minimal and never exceeds 5 ml per test.

*Coulter Electronics Inc, 590 West Twentieth Street, Hialeach, FL 33010, USA.*

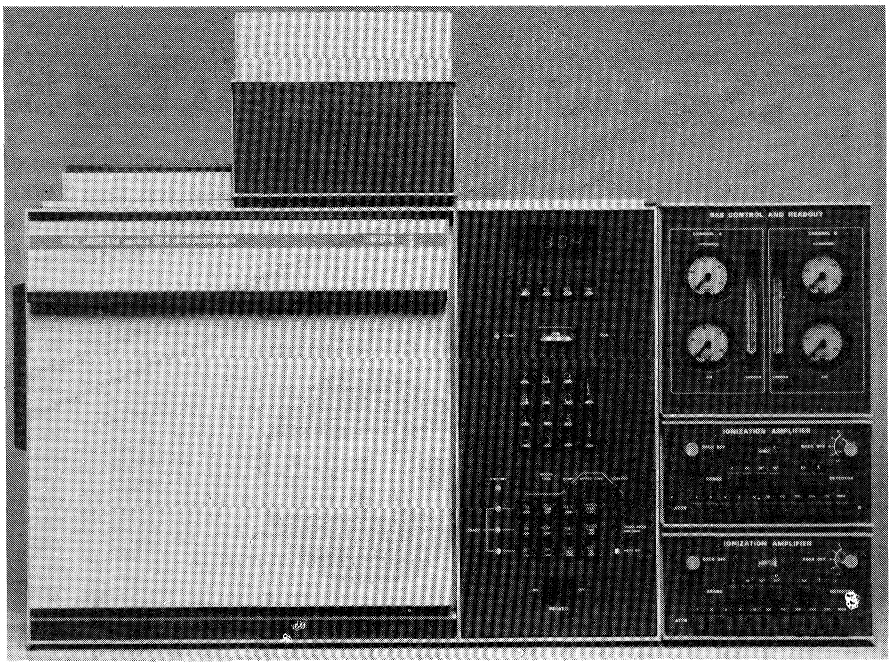

*Pye Unicam's microprocessor controlled gas chromatograph*

### Interactive data analysis for Du Pont 1090 thermal analysis/data system

An interactive data analysis capability has been added to Du Pont's 1090 Thermal Analyser to provide a totally integrated, third generation thermal analyser/data system. As such this produces the maximum useful information in the minimum time.

Introduced at the 1980 Pittsburgh Conference, the 1090 System consists of three major parts: the 1090 Temperature Programmer/Recorder, the 1091 Disk Memory, and the 1092 Data Analyser.

Heart of the 1090 system is the highly versatile programmer/recorder with an alphanumeric keyboard, printer-plotter, and bright plasma display panel for operator-instrument dialogue. Key benefits include 12 linkable program methods, improved temperature control, increased heating rate selection, sample temperature recording, linear scaling and all alphanumeric programming and recording.

While data analysis may be the last step in an experiment, it may be the first in importance. The differential scanning calorimetry (DSC) and dynamic mechanical analyser (DMA) programs provide rapid user interactive analysis of data stored in the 1091 disk memory. The DSC program, for example, measures glass transition temperatures and heats of both endothermic and exothermic events. The DMA program calculates and plots eight functions, including elastic modulus, loss modulus, and tan delta derived from the original experimental data.

Technically exciting as this state-of-the-art instrument is, its real importance lies in its ease of use and increased production of useful analytical information. Automatic report preparation is another important feature and full use is made of the recorder paper and the analyser scales and prints recorder axes within ranges selected by the operator. All conditions, including sample identification, technique, operator, and date can be labelled by the alphanumeric printer/plotter. The final publication-quality thermogram is printed on standard notebook-sized paper for easy filing or for direct insertion into reports or journal articles.

*Du Pont, Analytical Instruments Division, McKean Building-Concord Plaza, Wilmington, Del. 19898. USA.*

### Lipid controls

Helena Laboratories have announced the availability of four new lyophilized control serums. Lipotrol provides visual and quantitative separation of the alpha, pre-beta and beta lipoproteins for the electrophoretic techniques used to assess hyperlipoproteinemia. Three other controls for HDL cholesterol fractionation include their high, normal and low HDL cholesterol controls. Each of these controls is suitable for use with both the electrophoretic and heparin-manganese precipitation methods. Each control provides assayed values for HDL, LDL and VLDL fractions.

*Helena Laboratories, PO Box 752, 1530 Lindbergh Drive, Beaumont, Texas 77704, USA.*

### Gas chromatograph

A new microprocessor controlled gas chromatograph, designed for flexibility and ease of operation is now available from Pye Unicam. The Series 304 chromatograph has a keyboard with operating keys labelled in chromatographic terms, a full set of status lights and an ergonomic front panel layout. A full digital display gives information about the current state of the instrument and shows all set parameters and actual values. It highlights illogical entries and oversights as well as displaying diagnostics when necessary.

The operator is notified of any problems immediately because a built-in integrity check which occurs each time the unit is 'powered up' covers the display, status lights and memory. Rapid identification of any fault is achieved by the use of a signature analysis which pinpoints the fault location.

The instrument incorporates start-up/cool-down sequences which ensure that the detectors are maintained at a higher temperature than the injector and columns during heat-up and cool-down. There is a built-in temperature programmer which allows a three-level matrix temperature programme to be entered.

*Pye Unicam Ltd, York St, Cambridge CB1 2PX, UK.*

### Sulphur analyser reagents

A sulphur analyser titrant and diluent are available from Fisher Scientific. The reagents reduce reagent cost-per-test and eliminate waste disposal problems because the titrant can be recycled by adding small amounts of iodine. The reagents provide enhanced stability and repeatability and have a longer shelf-life. They can be used with all sulphur analyser models.

*Fisher Scientific Co, 711 Forbes Ave, Pittsburgh, PA 15219, USA.*

